# Dental ontogeny and replacement in Pliosauridae

**DOI:** 10.1098/rsos.150384

**Published:** 2015-11-04

**Authors:** Judyth Sassoon, Davide Foffa, Ryan Marek

**Affiliations:** 1 School of Earth Sciences, University of Bristol, Wills Memorial Building, Queen’s Road, Bristol BS8 1RJ, UK; 2 School of GeoSciences, Grant Institute, University of Edinburgh, The King’s Buildings, James Hutton Road, Edinburgh EH9 3FE, UK; 3 Department of Musculoskeletal Biology, University of Liverpool, Duncan Building, Daulby Street, Liverpool L69 3GE, UK

**Keywords:** CT scans, digital models, dental ontogeny, Pliosauridae, Sauropterygia, tooth replacement

## Abstract

Dental morphology and patterns of tooth replacement in representatives of the clade Pliosauridae (Reptilia, Sauropterygia) are evaluated in detail. The jaws of one basal (*Thalassiodracon hawkinsii*) and two derived species (*Pliosaurus* *carpenteri*, *Pliosaurus kevani*) were visualized by μCT scans, and the ontogenetic patterns, or ‘movement paths’, of replacement teeth could be mapped. Other specimens (*Peloneustes philarchus* and *Pliosaurus westbuyensis*) with well-preserved jaws containing functional and replacement teeth *in situ* were also examined directly, and waves of tooth replacement could be inferred from the degree of *in situ* tooth development and the fusion between functional and replacement alveoli. The analysis revealed symmetrical tooth eruption over the medial axis throughout the length of the jaw in the basal pliosaurid *Thalassiodracon*. By contrast, symmetrical tooth eruption patterns occur only along the anterior sections of the jaws of derived pliosaurids. In *Pliosaurus*, replacement schedules differ in the anterior and posterior portions of the jaws and appear to correlate with differences in tooth morphology and symmetrical replacement. The anterior teeth exhibit longer replacement cycle periods and symmetrical replacement, while shorter cycle periods and asymmetry are seen posteriorly. A longer period suggests slower replacement and is characteristic of large, specialized caniniform teeth in the longer snouted Late Jurassic taxa. Smaller posterior teeth have a shorter period and therefore a faster replacement cycle. The transition from long to short replacement period over the length of the jaw is thought to account for the loss of symmetry. This differentiation could relate to differential tooth function and a type of heterodonty. We therefore propose a new model of pliosaurid tooth replacement patterns and present it in a phylogenetic context.

## 1. Introduction

All reptiles, including extinct forms studied to date, renew their dentition continuously throughout life (polyphyodonty) [1,2]. Consequently, fossilized jaws sometimes contain teeth at different stages of development. Various attempts to understand patterns of tooth replacement have been made, and waves of replacement (Zahnreihen) were proposed as a characteristic feature of replacement in both extant and extinct reptiles [2–4]. The ‘Zahnreihen’ hypothesis was tentatively favoured by some authors [5–9] while others argued against it [10].

A general sequence of tooth replacement events has been suggested in reptiles, in which budding replacement teeth, lying in resorption pits, develop lingually to their corresponding functional teeth. As the new tooth develops, the attachment of the old functional tooth is weakened, the functional tooth is lost and the replacement tooth takes its place [1,4]. However, the process varies in rate, geometry and pattern in some reptile clades, such as varanid lizards and anguinomorph lizards [4,11], theropod and sauropod dinosaurs [12,13]. Importantly, these examples demonstrate the difficulties that occur when general rules are applied to excessively large phylogenetic brackets and they highlight the need for clade-focused studies. In Mesozoic marine reptiles such as Ichthyopterygia, replacement follows the general pattern just outlined [1], while in Eosauropterygia, there is a unique process in which teeth develop in distinct alveolar spaces (crypts) located distolingually to each functional tooth [14]. This is similar to the process described in mosasaurs [15], which independently evolved thecodonty from pleurodont ancestors.

To date, tooth replacement studies in Sauropterygia have focused on Triassic taxa such as Nothosauridae and Placodontia [14,16,17], and few detailed studies have been made of other subclades. The pliosaurs (Pliosauridae *sensu* Benson & Druckenmiller [18]) are one such group nested within the Sauropterygia, within Plesiosauria. They were a successful group of macropredatory marine reptiles and dominated the oceans from the Early Jurassic (Hettangian) [19] until the early Late Cretaceous (Turonian), attaining large body size and a large-headed, short-necked body plan [20,21]. Considering the significance of pliosaurids as top predators, information on their tooth ontogeny, maintenance and replacement is of some importance in understanding their feeding adaptations and ecological role.

Individual pliosaurid teeth have been described and figured in detail since the earliest days of palaeontology. The eponymous genus *Pliosaurus* [22] includes Late Jurassic pliosaurids on the basis of teeth with a trihedral cross section [22–24] (table 1) and the holotype of *Liopleurodon ferox* is a single, large tooth [25]. Also tooth durability has made them important elements in recording pliosaur occurrences in the absence of more complete remains. However, apart from mentions in descriptive contexts [26–28] and a detailed study of ‘*Liopleurodon macromerus*’ [29], the patterns and dynamics of tooth development and replacement have not been studied. This is largely because of the paucity of pliosaurid specimens preserving sufficient teeth *in situ*, and the technical difficulties associated with studying the internal anatomy of tooth replacement.

**Table 1.** Tooth morphology.

specimen	geological provenance	tooth cross section	tooth morphology
*Thalassiodracon hawkinsii* (CAMSM J.46986)	Rhaetian/Hettangian, Lower Jurassic, Blue Lias Formation	suboval	slender, weakly curved, apicobasally oriented ridges, some extending to apex of crown
*Liopleurodon ferox* (NHMUK R2680)	Callovian, Middle Jurassic, Oxford Clay Formation	subcircular	strongly curved, coarsely ornamented with ridges enlarging towards apex. Few ridges reach apex convex surface variably ornamented
*Simolestes vorax* (NHMUK 3319)	Callovian, Middle Jurassic, Oxford Clay Formation	subcircular	slender, weakly curved, fine ridges on enamel, crescent shaped region of unridged enamel at base of crown, tightly packed ridges on concave surface
*Peloneustes philarchus* (NHMUK R8574)	Peterborough Member, Callovian, Middle Jurassic, Oxford Clay Formation	subcircular	distally recurved, conical, enamel ornamented with longitudinal ridges, originating from base of enamel, most ridges extend over half the apicobasal height of enamel, few reach the apex. Greater spacing of ridges on convex, mesial surface
*P. kevani* (DORCM G.13,675)	Upper Kimmeridgian *A. mutabilis* Biozone	sub-trihedral	conical, curved teeth with flattened labial surface. Coarse, apicobasally oriented ridges. No ridges on flattened, mesial surface
*P. westburyensis* (BRSMG Cc332)	Upper Kimmeridgian *A. eudoxus* Biozone	fully trihedral	conical, curved teeth with extremely flattened, ridgeless mesial surface
*P. carpenteri* (BRSMG Cd6172)	Upper Kimmeridgian *A. eudoxus* Biozone	fully trihedral	conical, curved teeth with extremely flattened, ridgeless mesial surface

Here we characterize tooth replacement anatomy and ontogeny in several pliosaurid specimens. This paper incorporates morphological and developmental data from five genera representing a time span from the basal Early Jurassic pliosaurid *Thalassiodracon* to the derived Upper Jurassic giants, represented by *Pliosaurus* (see the electronic supplementary material, figure S1). Synthetic studies of this kind are rare, perhaps because it has been assumed that there is a uniform pattern of tooth replacement within the Plesiosauria, with no additional information to be gained from studying different species. However, even within the Pliosauridae, we found both homodonty and heterodonty. In this paper, we attempt to correlate tooth size and shape with replacement patterns. Using μCT scans in combination with direct specimen observation, we demonstrate the detailed anatomy of tooth insertion, study the replacement patterns and tackle the question of ‘heterodonty’ in this clade.

## 2. Material and methods

Dentitions in basal and derived representatives of Pliosauridae were examined. Specimens include the basal pliosaurid *Thalassiodracon hawkinsii* (CAMSM J.46986) [19], NHM R2039 (lower jaw only); *Peloneustes* NHMUK R8574 [30]; the derived giant Upper Jurassic pliosaurids *Pliosaurus westburyensis* (BRSMG Cc332) [26], *Pliosaurus carpenteri* (BRSMG Cd6172) [27] and *Pliosaurus kevani* DORCM G.13,675 [31]. Two other specimens, *Simolestes* *vorax* (NHMUK R3170) and *Liopleurodon ferox* (NHMUK R3536), were also compared for tooth replacement symmetry but their preservation did not allow for detailed studies.

Internal and external tooth replacement anatomy was reconstructed from gross morphology studies and μCT scans. Replacement symmetry was assessed by correlating the degree of tooth development in corresponding alveoli, from the left and right tooth margins of upper or lower jaw. The ontogenetic stage of the teeth was assessed from tooth size, its degree of protrusion from functional alveoli and the degree of fusion between primary and secondary alveoli. Filled-in replacement alveoli were associated with newly emerged mature teeth and the newly forming bud of the next tooth cycle. The cross-sectional areas of empty functional alveoli (tooth sockets) were found to be a good proxy for the final size of the tooth they were to accommodate. Thus, it was possible to determine the distribution of large and small teeth in tooth arcades, even when teeth were not preserved *in situ*.

Computed Tomography (CT) scans were obtained from BRSMG Cd6172 and DORCM R3170. A custom built Nikon 450 kV micro-focus X-CT system (at the ‘μ-VIS’ Centre for Computed Tomography, University of Southampton) was used, configured as follows: transmission (approx. 1 μm spot limit, low flux); standard reflection (approx. 3 μm spot, ‘normal’ flux); rotating target (approx. 10 μm spot limit, x3–5 flux); 2×2 k flat panel detector; samples to approximately 300 mm and 50 kg; robotic sample exchange (approx. 150 mm height limit). The digital modelling and processing was made with the 3D analysis software Avizo® 6.1 and 6.3 at the University of Bristol. Scans for CAMSM J.46986 were obtained from Dr. Roger Benson, University of Oxford [19].

Bar graphs showing tooth replacement cycles were created by visually approximating developmental stages and abstracting them onto bar lengths on charts. Maximum bar lengths represent the maximum sizes reached by teeth, irrespective of their actual mature length in the tooth arcade, so there is no absolute quantitative correlation between bar length and tooth length. The approximation of the stages of one tooth cycle was made by assessing one cycle period, defined as the number of alveoli between matching tooth development stages along a tooth arcade. The sequence of sizes of tooth development stages in between matching stages was then used to characterize the divisions of one cycle.

### 2.1 Institutional abbreviations

BRSMG, Bristol City Museum and Art Gallery, Bristol, UK; CAMSM, Sedgwick Museum of Earth Sciences, Cambridge University, Cambridge, UK; DORCM, Dorset County Museum, Dorchester, UK; NHMUK, Natural History Museum, London, UK.

## 3. Results

### 3.1 Tooth descriptions

**Figure 1.**
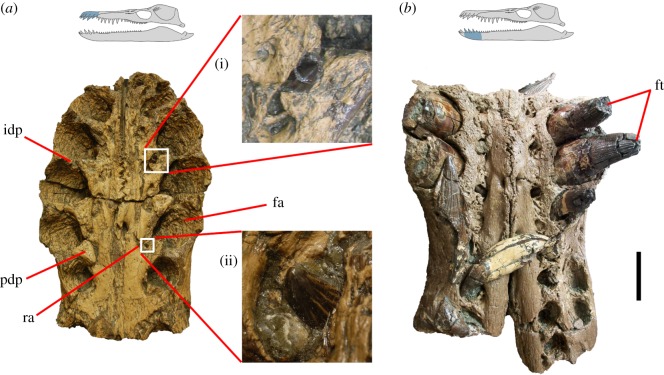
(*a*) Ventral view of premaxilla in *P. carpenteri* (BRSMG Cd6172) showing magnified images (i) and (ii) of replacement teeth emerging between paradental plates. (*b*) Dorsal view of a section of dentary, showing replacement alveoli. Replacement teeth do not restructure the bone surface of the dentary. fa, functional alveolus; ft, functional tooth; idp, interdental plate; pdp, paradental plate; ra, replacement alveolus. Scale bar, 10 cm.

**Figure 2.**
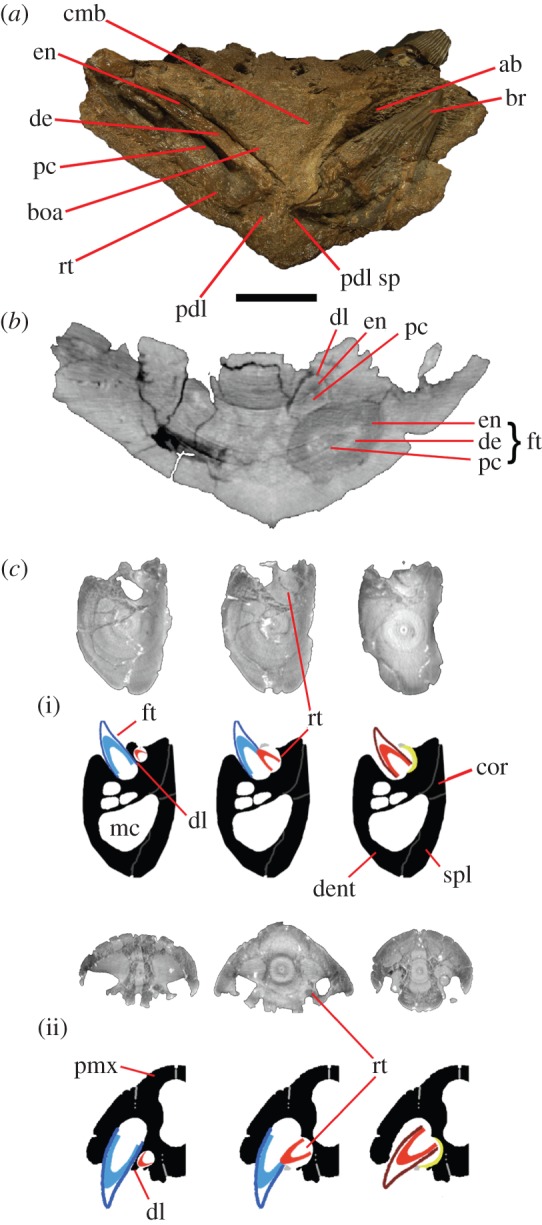
Anatomy of tooth replacement in pliosaurids. (*a*) Cross section through *P. carpenteri* (BRSMG Cd6172) dentary in posterior aspect at level of the fourth alveoli. Section of complete caniniform tooth visible *in situ* on left. (*b*) μCT scan slice of *P. carpenteri* showing orientation of replacement caniniform tooth with respect to functional tooth. (*c*) Tooth replacement sequence in (i) dentary and (ii) premaxilla in pliosaurids based on μCT scan slices from *P. kevani* (DORCM G.13,675). Blue, mature tooth; red, replacement tooth. ab, alveolar bone; br, broken tooth collapsed in alveolus; boa, bone of attachment; cmb, compact mandibular bone; cor, coronoid; de, dentine; dent, dentary; dl, dental lamina; en, enamel; ft, functional tooth; mc, Meckel’s canal; pc, pulp cavity; pdl, periodontal ligament; pdl sp, periodontal space; pmx, premaxilla; rt, replacement tooth; spl, splenial. Scale bar, 10 cm.

Pliosaurid teeth are monocuspid, with an elongate, cylindrical base capped by a projecting, conical crown. The main body of the tooth consists of a smooth layer of calcified dentine secreted by odontoblasts, as in other vertebrates [32]. The upper portion of the dentine is covered by enamel, ornamented by asymmetrically distributed apicobasal ridges. Ridges vary in number, coarseness and extent in different pliosaurid species, and can encompass the whole circumference of the tooth. Coronal ornamentation is restricted to the enamel. The teeth vary in shape and ornamentation (sometimes of diagnostic importance) across the clade. Ridges in the caniniform teeth of large pliosaurids are subtriangular in cross section and raised, forming an irregularly roughened surface. In the sub-trihedral or trihedral teeth of *Pliosaurus*, ridges are absent from the labial enamel surface (figure 1). The proportion of dentine to enamel varies in different teeth within each specimen. At the base of the tooth, dentine surrounds an open pulp cavity, which extends beyond the base of the enamel but does not reach the top of the crown. In life, the cavity was presumably rich in fibroblast cells, blood vessels and nerves, but none of the soft tissues is preserved and a hollow, or infilled cavity is left behind. In unbroken teeth, a circular ridge encircles the pulp cavity at the base of the dentine (figure 2). Individual teeth from pliosaurids have been described in the literature [19,26,27,30,31] and a comparative summary is provided here (table 1).

### 3.2 Dental anatomy, attachment and ontogeny

#### 3.2.1 Tooth implantation and attachment

**Figure 3.**
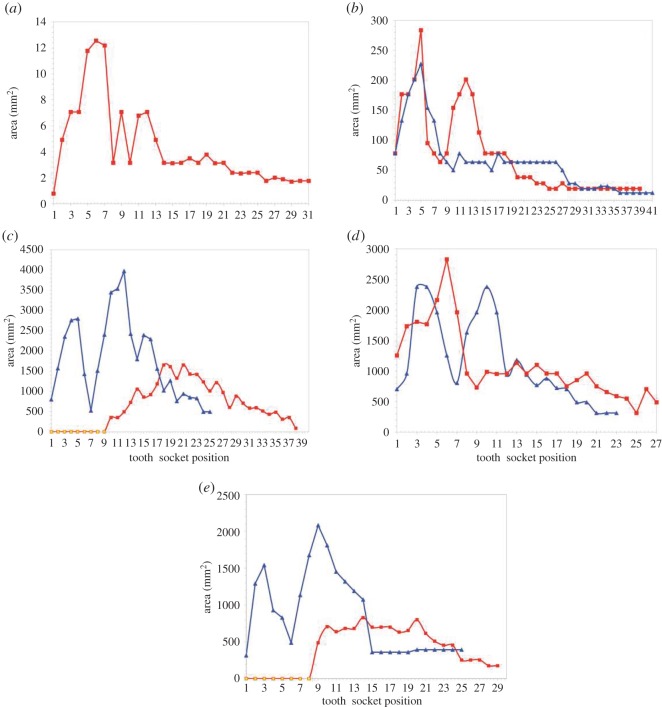
Graph of alveolar size expressed as area (mm^2^) in (*a*) *Thalassiodracon* (NHMUK R2039), (*b*) *Peloneustes* (NHM R8574), (*c*) *P. kevani*, (*d*) *P. carpenteri* and (*e*) *P. westburyensis*. Blue line, upper jaw; red line, lower jaw; yellow squares, alveoli in missing mandibular symphysis, as inferred by Benson *et al*. [34].

In pliosaurids, tooth attachment is thecodont, i.e. teeth are rooted in deep alveoli lined with a layer of alveolar bone (figures 1 and 2). The roots are supported by the periodontium, consisting of complex, uncalcified, soft connective tissue fibres embedded at one end in the alveolar bone and at the other in the cement coating the tooth surface (Sharpey’s fibres [15]). Most large pliosaurid skulls do not preserve mature teeth *in situ* because they fall out following post-mortem degradation of periodontal ligaments [33]. The majority of empty alveoli in the specimens studied do not contain any tooth remnants, so teeth were mostly not broken but lost in their entirety. This kind of tooth loss is rare in tetrapods with ankylosed teeth and is the evidence for soft periodontal connections between teeth and alveoli. By contrast, immature, developing teeth may be retained in replacement alveoli because, even though their periodontal tissue has given way to taphonomic disintegration, the surrounding bone keeps them securely in place. Our studies show that the size of alveoli is a good proxy for the size and type of mature tooth they are destined to hold (figure 3).

The alveoli are separated by interdental plates (figure 1). Paradental plates are present lingual to the alveoli. In more derived pliosaurids, paradental plates are large and triangular and partially constructed from alveolar bone, as in most vertebrates [35,36]. Alveolar bone is resorbed when a tooth is shed and redeposited when a new tooth develops. Alveolar bone typically lacks the Haversian system and appears rough and unstructured, reflecting the non-parallel orientation of collagen fibres on which it forms. Large, well-developed paradental plates are characteristic of derived, Late Jurassic, giant pliosaurs (figure 1).

#### 3.2.2 Tooth ontogeny in pliosaurids

Here, we show for the first time the ontogenetic pattern, or ‘movement path’, of pliosaur replacement teeth using μCT scans. The scans show that at inception a replacement tooth starts off recumbent and becomes more vertical during development. Alveoli accommodating the largest caniniform teeth are extraordinarily deep and their roots occupy a considerable part of the jaw volume (figure 2). By contrast, alveoli accommodating the posteriormost teeth are very shallow and the question of whether the smallest posteriormost alveoli could actually house teeth at all has been raised [26].

Replacement tooth germs form in crypts dorsomedial (mandible) and ventromedial (upper jaw) to the functional alveoli (figures 1 and 2). Replacement alveoli generate and develop within a groove, disto-medial to their corresponding functional alveoli. They are also initially separate from the functional alveoli. During tooth ontogeny, the bone separating each functional alveolus and the replacement alveolus becomes progressively resorbed as the replacement tooth grows and moves labially, entering the functional alveolus. This process is known as alveolarization [4,14]. The apex of the new tooth becomes exposed on the dentigerous bone before erupting onto the surface and the older functional tooth becomes loose by root resorption and drops out. The new tooth then develops to maturity within the functional alveolus. Dentigerous bone partially seals the replacement alveolus, so it is possible to identify alveoli with recent replacements, even if teeth are not preserved *in situ*. This description and the μCT scans describe processes in the anterior caniniform teeth which lie in a rather procumbent orientation. The more vertically oriented posterior teeth are replaced as suggested by Rieppel [14].

### 3.3 Heterodonty in pliosaurids

**Figure 4.**
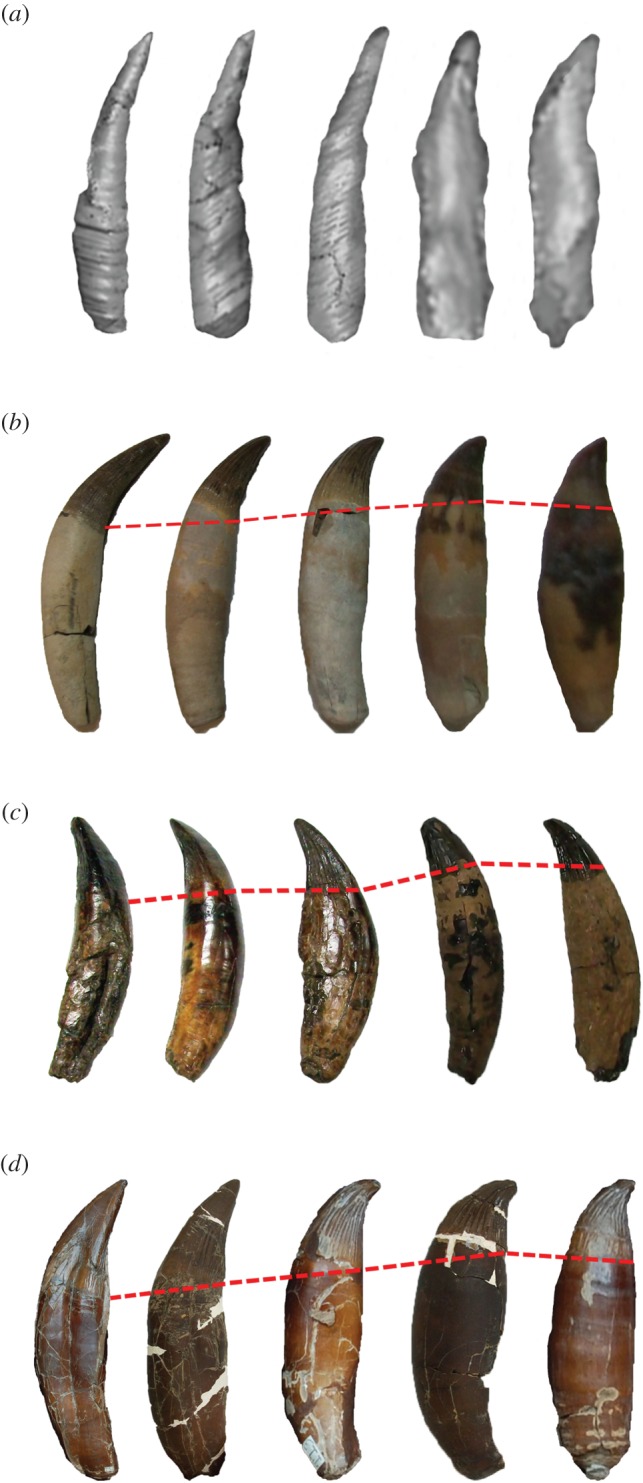
Pliosaurid teeth from different regions of the jaw scaled to same height to highlight differences in tooth morphology. Red line differentiates crown from root. (*a*) *Thalassiodracon hawkinsi* (CAMSM J.46986) teeth from μCT scan digital model. I, II, III anterior teeth, IV, V posterior teeth; (*b*) *Peloneustes* sp. (NHMUK R8574). I, II anterior teeth III, IV, V posterior teeth; (*c*) *Pliosaurus westburyensis* (BRSMG Cc332) I, II anterior caniniform teeth, III, IV, V posterior teeth; (*d*) *Pliosaurus carpenteri*(BRSMG Cd6172) I, II, III anterior caniniform teeth, IV, V posterior teeth. Not in scale.

We measured and analysed the degree of tooth variation along the jaws of the basal pliosaurid *Thalassiodracon*, and two derived genera, *Peloneustes* and *Pliosaurus* (figure 4). *Thalassiodracon hawkinsii* is a small basal pliosaurid, with a long-necked, ‘plesiosauromorph’ [20] body plan. Its pliosaurid affinity was established by Benson *et al*. [19] (see also the electronic supplementary material, figure S1). The skull is relatively small and proportionally short (table 2), housing numerous slender teeth. All other specimens in this study had a more typical ‘pliosauromorph’ body plan with higher head to neck ratios and longer skulls. Our analysis assessed whether dentition is uniform (excluding variation in size) or if there is a distinct morphological variation.

**Table 2.** Comparison of skull proportions in pliosaurids.

specimen	ratio of antorbital skull length to total skull length
*Thalassiodracon hawkinsii* (CAMSM J.46986)	0.47
*Peloneustesphilarcus* (NHMUK R8574)	0.64
*Simolestes vorax* (NHMUK 3319)	0.53
*Liopleurodon ferox* (NHMUK R2680)	0.58
*Pliosaurus westburyensis* (BRSMG Cc332)	0.55
*Pliosaurus carpenteri* (BRSMG Cd6172)	0.57
*Pliosaurus kevani* (DORCM G.13,675)	0.59

Tooth shapes in the anterior and posterior regions of the jaws of two derived genera, *Peloneustes* and *Pliosaurus* show considerable variation in overall shape (figure 4) and curvature (table 3). In all pliosaurids more derived than *Thalassiodracon*, the calculated curvature of the anterior caniniform teeth and the posteriormost teeth did not vary by much, but the stoutness of the posterior teeth was significantly greater (table 3).

**Table 3.** Tooth statistics for pliosaurids. The tooth curvature was the measured proportion of concave length to convex length of a complete tooth. Stoutness was the measured proportion of the largest circumference of the tooth to the length of a complete tooth. Data are presented as a mean value (*n*=4).

species	tooth type	crown length : tooth length	curvature	stoutness
*Thalassiodracon*	caniniform	0.40	0.93	0.49
	posterior	0.32	0.94	0.70
*Liopleurodon*	caniniform	0.36	0.85	0.59
	posterior	0.35	0.82	0.75
*Simolestes*	caniniform	0.34	0.90	0.40
	posterior	0.33	0.98	0.80
*Peloneustes*	caniniform	0.33	0.86	0.58
	posterior	0.23	0.82	0.78
*P. westburyensis*	caniniform	0.36	0.84	0.63
	posterior	0.30	0.82	0.85
*P. carpenteri*	caniniform	0.38	0.85	0.63
	posterior	0.31	0.81	0.76

Further evidence of tooth variation comes from the distributions of tooth sizes (expressed as the cross-sectional area of the corresponding alveoli) along the length of the upper and lower jaws of the specimens. All the examined taxa display a clear regional division into larger anterior teeth, with considerable size variation, and smaller posterior teeth, whose size decreases posteriorly with less variation. In *Pliosaurus*, there are two clear expansions on the upper jaw, the first being the premaxillary expansion, which accommodates six teeth, and the second being the maxillary expansion, which accommodates variably seven or eight caniniform teeth. Behind this expansion, the teeth become smaller and hooked, as described by Taylor & Cruickshank [26] for *P. westburyensis* and shown here (figure 4). This argues a case for a true heterodonty in derived pliosaurids, on the basis of size, shape (stoutness) (table 3) and regional partitioning (the stouter teeth are the smaller, posterior teeth), whereas in the basal pliosaurid there is regional partitioning of size but no real variation in shape (anisodonty).

We recognize that the distinction between heterodonty and anisodonty is somehow arbitrary and that the two terms are likely to represent the end members of a continuum, but in this paper we adopt both a morphological and physiological (see next §3.4) definition of heterodonty.

### 3.4 Tooth replacement schedules in pliosaurids

Tooth replacement patterns are described for four pliosaurid taxa, *Thalassiodracon*, *P. carpenter*i, *P*. *kevani* and *Peloneustes*. μCT scans were available for *Thalassiodracon* and the two *Pliosaurus* specimens. Tooth replacement schedules for *Peloneustes* were inferred by detailed observation of both upper and lower jaws. Only the lower jaw had sufficiently well preserved teeth to infer tooth cycle patterns.

**Figure 5.**
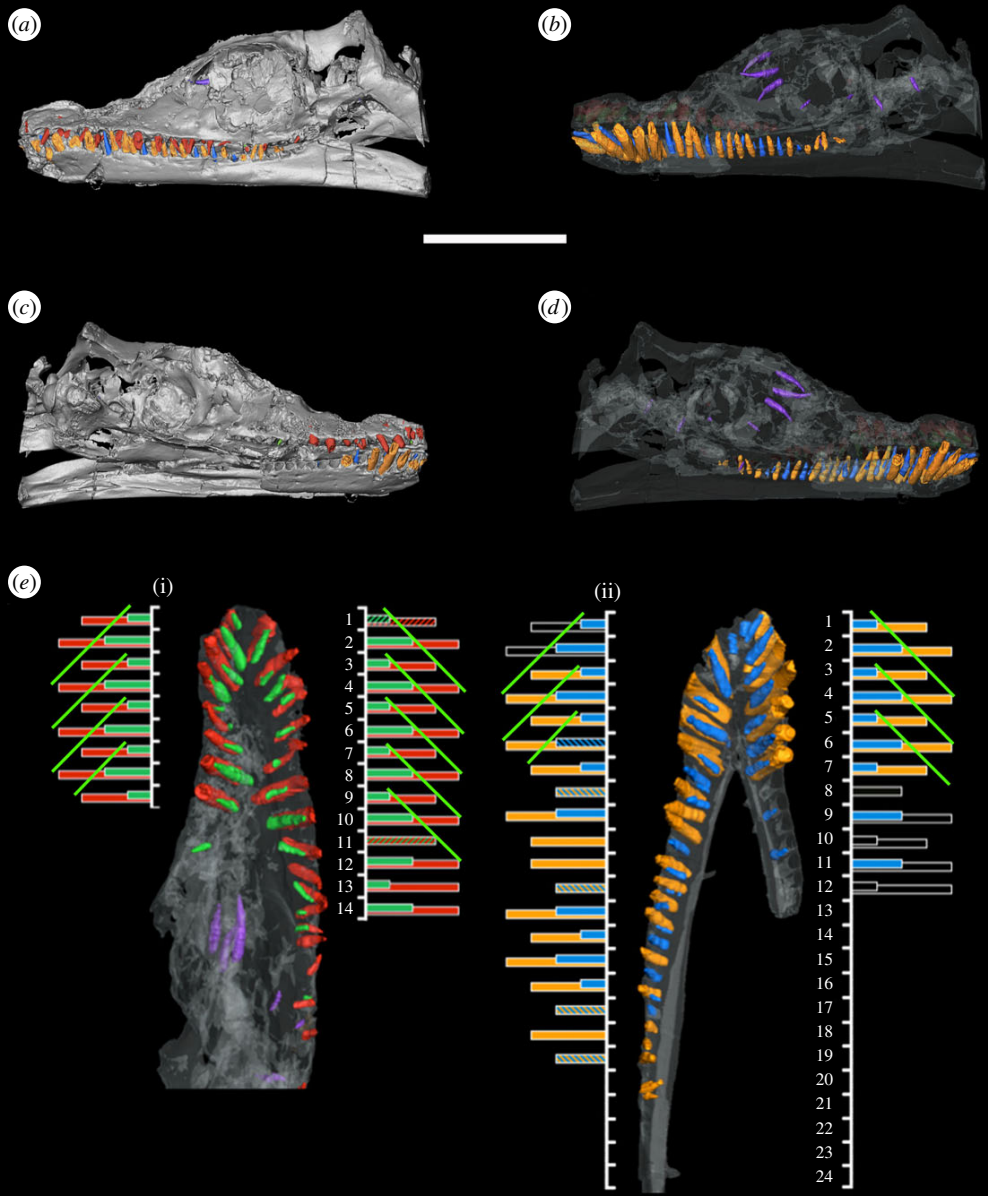
Digital models of tooth replacement in *Thalassiodracon hawkinsi* (CAMSM J.46986). (*a*,*b*) solid, (*c*,*d*) transparent reconstructions of skull in lateral view. (*e*) Transparent reconstruction of (i) upper jaw and (ii) mandible, with corresponding tooth replacement cycle histograms. The height of bars corresponds to tooth maturity. Diagonal lines (green) indicate replacement period of 2. Upper jaw: mature teeth, red; replacement teeth, green. Dentary: mature teeth, yellow; replacement teeth, blue; loose teeth, purple. Scale bar, 5 cm.

In *Thalassiodracon*, μCT scans showed fully mature thecodont teeth implanted in deep alveoli that make up much of the jaw volume (figure 5). Developing tooth germs lying in crypts were visible through foramina (replacement alveoli) oriented dorsomedial and slightly posterior to the mature teeth. The histogram (figure 5*e*) shows tooth replacement cycles. Tooth replacement is symmetrical across the medial axis (*contra* [4]) with teeth at the same stage of development appearing in corresponding alveoli across the medial axis. Replacement and functional teeth are distinguished by colour (as outlined in the figure legends). One replacement cycle can be broadly divided into four steps, the first two of which represent replacement teeth maturing in replacement alveoli, while their corresponding functional teeth are still in place. The final two stages represent the teeth maturing in the corresponding functional alveoli following the displacement of the previous tooth. The replacement takes place in cycles as proposed by Edmund [4], which produce repeating wave-like patterns along the jaw. The green coloured lines show the waves for *Thalassiodracon* (figure 5*e*). They are illustrated here as four-stage waves, with a period of 3 (in other words, at every third tooth, a new wave begins). In *Thalassiodracon*, the pattern continues smoothly across the premaxillary–maxillary boundary.

**Figure 6.**
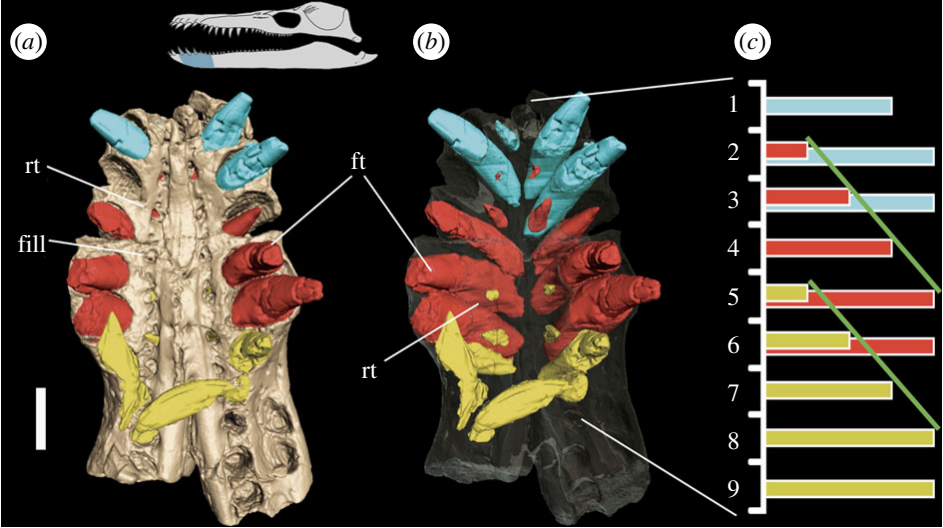
(*a*) Solid and (*b*) transparent reconstructions of the symphysial region of *P. carpenteri* (BRSMG Cd6172). (*c*) corresponding histogram showing caniniform tooth replacement cycles. Diagonal lines (green) indicate replacement period of 3. Colours (blue, red, yellow) indicate three different tooth cycles. ft, functional teeth; rt, replacement teeth; fill, filled in alveolus. Scale bar, 5 cm.

A similar pattern is seen in *P. carpenteri* in which only the symphyseal region could be μCT scanned (figure 6). The tooth replacement cycles are symmetrical across the medial axis, as in *Thalassiodracon*, but differ in that they have a longer period of 4. The cycles have been reconstructed into five stages (figure 6*c*), the first two stages representing replacement teeth maturing alongside their corresponding functional teeth, while the last three stages are teeth maturing in the functional alveoli. Two complete cycles and the last three stages of a third could be reconstructed (figure 6*c*).

**Figure 7.**
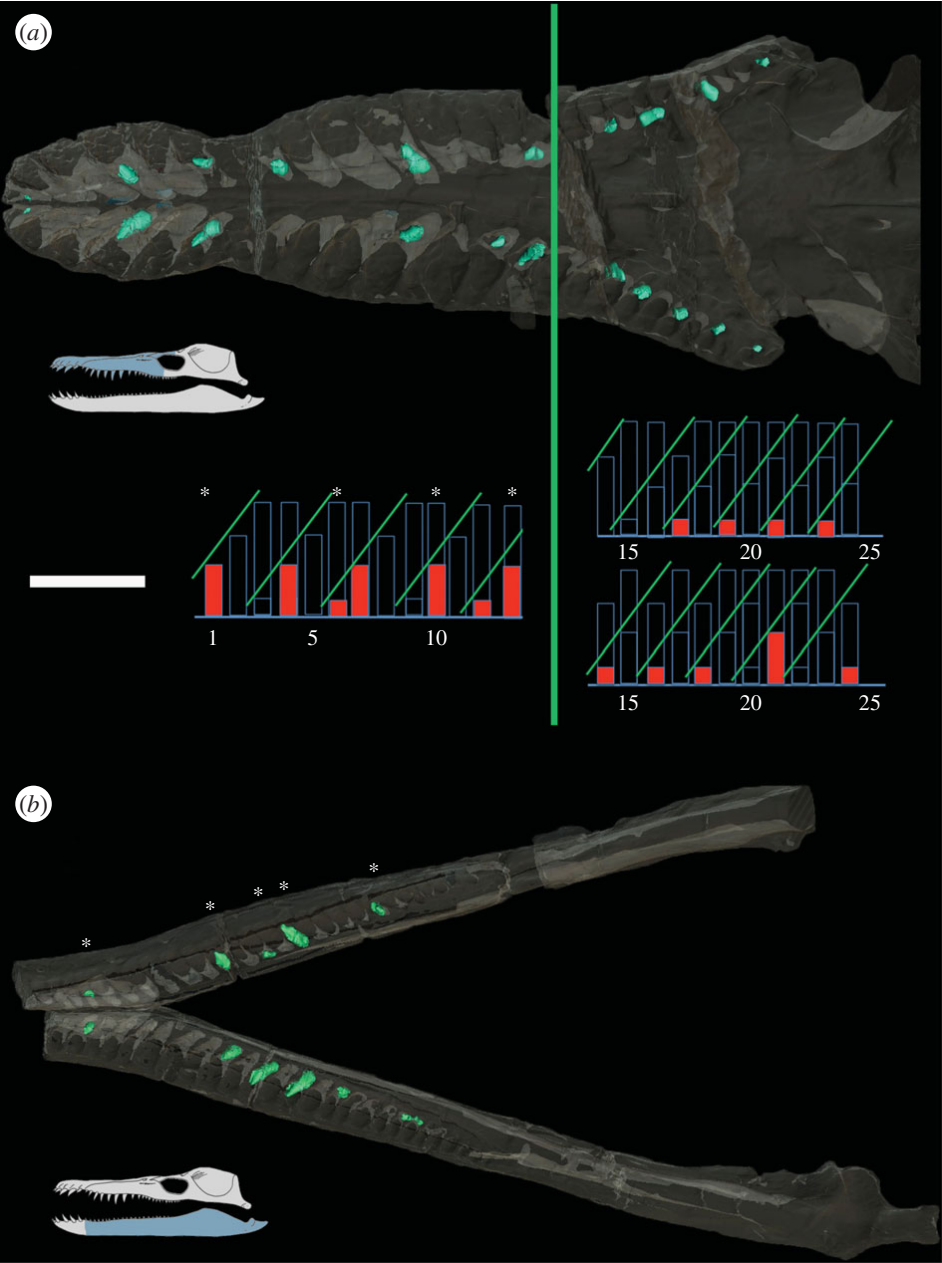
Transparent digital models from μCT scans showing tooth replacement in (*a*) upper jaw and (*b*) lower jaw of *P. kevani.* Replacement teeth shown in green. Functional teeth were not preserved *in situ* but stage of missing tooth development could be inferred from the degree of fusion between functional and replacement alveoli. (*a*) Histograms show inferred tooth replacement cycles in the upper jaw. The single chart showing bars for alveoli 1–13 is the combined results from left and right jaws. Asterisk (*) indicates teeth preserved symmetrically on right and left side. No fill bars show the inferred presence and developmental stage of a tooth. Red fill indicates a tooth is preserved on either left or right. The two charts for cycles beyond alveolus 14 are for the left side (above) and right side (below). Period decreases to 2 on both sides and a shift occurs in the left jaw, so symmetry is lost. (*b*) A histogram was not plotted for the mandible, but symmetrical tooth preservation on the right and left dentary can still be seen (*). Symmetrical teeth are at the same stage of development. The apparently small tooth in socket 14 on right dentary is a broken mature tooth. Scale bar, 10 cm.

In *P. kevani*, there were no mature teeth preserved in the alveoli. However, 34 replacement teeth were preserved and clearly visible in μCT scans (figure 7). The stages of tooth development could also be inferred from the degree of fusions between functional and replacement alveoli. On the assumption that tooth replacement was probably symmetrical (as in *Thalassiodracon* and *P. carpenteri*), replacement cycles were inferred using teeth preserved from both left and right tooth arcades. With this method, it was possible to fit the preserved premaxillary and early maxillary teeth into cycles of period 4 (as in *P. carpenteri*). However, beyond alveolus 16, there is a change both in period and symmetry. The cycles appear to pack more closely, the period decreasing to 2, and symmetry is lost. The change in replacement rhythm produces a shift in the pattern of the left maxilla beyond alveolus 16, such that cycles in left and right maxillae are out of phase posteriorly.

**Figure 8.**
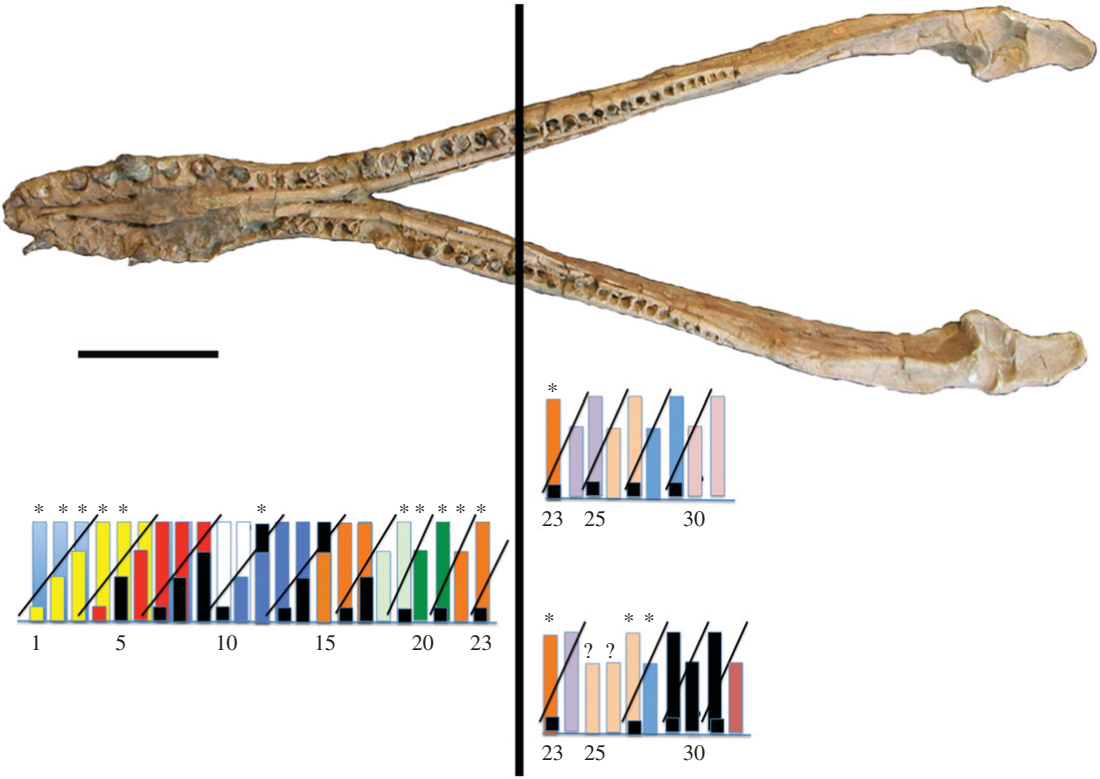
Tooth replacement in mandible of *Peloneustes* sp. (NHMUK R8574), with corresponding histograms plotted for alveoli 1–31 (right) and 1–32 (left) mandible. Single histogram for anterior portion of mandible shows inferred tooth replacement cycles between alveoli 1 and 23. Mature teeth (*blue*) in alveoli 1–3 are endpoints of two previous replacement cycles. Different colours show successive tooth replacement cycles. Asterisk (*) indicates teeth preserved symmetrically on right and left dentary. *Black fill* shows inferred tooth, not preserved or not visible. The replacement period for teeth in alveoli 1–16 is 3. Beyond alveolus 16, period decreases to 2. Symmetry breaks down at alveolus 24. The pair of histograms show replacement cycles after breakdown of symmetry, in right (above) and left (below) mandibular ramus. The replacement rhythm breaks down in the left ramus at alveolus 25 and then is restored. Symmetry might be restored after alveolus 27. Scale bar: 50 cm.

CT scans were unavailable for *Peloneustes* (NHMUK R8574), a specimen preserving numerous teeth *in situ* in the lower jaw. Tooth replacement stages were assessed as before, from the presence of visible teeth in functional and replacement alveoli, and from the degree of alveolar fusion (figure 8). As with *P. kevani*, replacement symmetry is maintained anteriorly but in this specimen it breaks down at the 23rd alveolus. The breakdown of symmetry is also associated with a significant reduction in tooth size (figure 8). The period of the replacement cycles in *Peloneustes* is 4 up to alveolus 16, where the period drops to 3 to correspond with the more rapid replacement rhythm characteristic of smaller teeth (figure 8).

## 4. Discussion

We have shown that tooth shape, structure, enamel ornamentation and size evolve within the Pliosauridae. By contrast, the mode of implantation, attachment and the basic process of tooth replacement do not change either in this taxon or over the entire clade Plesiosauria. This is different from other marine reptile clades. For example, Maxwell *et al*. [1] observed variations in the general thecodont implantation within the Ichthyopterygia. By contrast, taxa within Sauropterygia tend to exhibit classical thecodonty throughout, where each tooth is placed within a deep socket and affixed by uncalcified connective tissue. Mosasaurs, by contrast, exhibit a secondary thecodonty derived from an ancestral lepidosauromorph pleurodont condition, and consequently have developed a unique ‘movement path’ of tooth replacement, different from the Sauropterygia [15]. Mosasaur tooth replacement is a ‘conveyor belt’ type alveolar migration, involving a large amount of mineralization (cementum). Not all the tooth crowns become attached and alveolar migration and tooth growth are separate processes, while in pliosaurids growth, alveolar migration and alveolar fusion are continuous (figure 2).

Different hypotheses of phylogenetic relationships among pliosaurids have been suggested [20,34,37–41]. In *Thalassiodracon*, a basal plesiosauromorph pliosaurid according to the scheme suggested by Benson & Druckenmiller [18], we observed a more homogeneous, anisodont dentition, while a significant degree of tooth differentiation could be seen in the derived genus *Pliosaurus*. We argue that the difference in teeth is sufficient for the term ‘heterodont’ to be used when describing *Pliosaurus* dentition. This heterodonty is defined on the basis of size (figure 3), shape (figure 4 and table 3), replacement rhythm and putatively function.

The teeth in *Pliosaurus* fall broadly into two kinds. The anterior set are deep-rooted, distally recurved caniniform teeth with a clear triangular cross section (trihedral), as in *P. westburyensis* and *P. carpenteri* [24,26,27], or a sub-trihedral cross section, as in *P. kevani* [31]. This caniniform set is anisodont, and varies in size only. These teeth are found in the premaxilla and the anterior, expanded portion of the maxilla (figure 1). The posterior teeth are a series of successively smaller, sharply recurved, hooked teeth with a more rounded cross section. In *P. kevani* there are no preserved posterior teeth, so it is not possible to be sure that they exhibit heterodonty like *P. carpenteri* and *P. westburyensis*, although it is likely that they do and a heterodont dentition is very probably a synapomorphy of *Pliosaurus*.

It seems likely that different growth control mechanisms operated in the replacement of caniniform and posterior teeth for such morphological differences to occur. The shift in replacement periods coincides with a significant reduction in tooth size in both *Pliosaurus*and*Peloneustes* (figures 7 and 8), while these small teeth become more hooked and stouter (table 3). However, the limitations of preservation preclude any concrete statements on the spatio-temporal expression of different odontogenic tissues [42].

We have shown that replacement tooth cycles in the basal pliosaurud *Thalassiodracon* are constant throughout the jaw and symmetrical over the medial axis (figure 5). By contrast, the more derived pliosaurid forms have longer cycle periods anteriorly and shorter cycle periods posteriorly (figures 6 and 7). A longer period suggests slower replacement and is characteristic of large, specialized caniniform teeth in the longer snouted Late Jurassic taxa. Smaller posterior teeth have a shorter period and therefore a faster replacement cycle. It was also observed that the shift from one cycling period to another sometimes affects the symmetry of tooth replacement in the posterior region of the jaws. This was observed in two specimens, *Pliosaurus kevani* and *Peloneustes,* and may occur more generally within Pliosauridae.

In functional terms, it has been suggested that the posterior hooked teeth of *P. westburyensis* acted as ratchets to aid in manoeuvring prey towards the gullet following capture [26], while the premaxillary and maxillary caniniforms had a powerful piercing function, characterizing the Pierce II/General Guild of Massare [43]. Edmund [2] mentioned that the replacement rhythm in reptiles was probably not maintained over the premaxillary–maxillary suture, but this is not what we observe here (figures 7 and 8). We suggest that synchronous tooth replacement acts across the premaxilla and maxilla in Pliosauridae, such that the entire set of caniniform teeth is replaced in a coordinated way, which is in line with the hunting and defending functions of these teeth. The symmetrical replacement of teeth was unexpected, as asymmetrical replacement in sauropterygians has been proposed elsewhere [16,28,29], although some symmetry was also observed in placodonts [17]. It would appear in that case that the loss of fangs on both sides at once had no detrimental effect on the hunting habits of pliosaurids.

Tooth insertion, replacement and morphology in conjunction with the skull structure and jaw musculature are responsible for the generation of bite forces during feeding. Functional analysis of bite forces in pliosaurids revealed that they were able to initiate high bite forces, but that the skull was structurally weak [44–46]. This was proposed as a trade-off between agility, hydrodynamics and strength [45], so pliosaurs probably did not restrain and shake their prey above the water surface, as modern crocodiles do, but more likely pursued, captured and swallowed food underwater. The comparative weakness in the skull construction would benefit ‘pursuit predator’ behaviour, as strongly built, heavy jaws would be more difficult to open and shut underwater. In considering this, symmetrical tooth replacement would not diminish the effectiveness of prey capture involving initial piercing followed by successive jaw snaps and ‘gulps’ as food was drawn into the gullet. One reason is that when the older tooth is lost, the newer tooth is already well developed and entering the functional alveolus (figure 2). Thus, the overall solidity of the jaw is not compromised, while there are functional piercing teeth positioned both anteriorly and posteriorly to the lost caniniform, which perform the required function.

## Supplementary Material

Supplementary Figure 1.

## Data accessibility

All data are available within the text or in the electronic supplementary material provided.
