# Supplementary material for: Dental ontogeny and replacement in Pliosauridae
Source: R Soc Open Sci. 2015 Nov 4;2(11):150384. doi: 10.1098/rsos.150384 (PMC4680613; doi:10.1098/rsos.150384)
Supplement: Supplementary Figure 1. [file rsos150384supp1.docx]

**Supplementary material.**

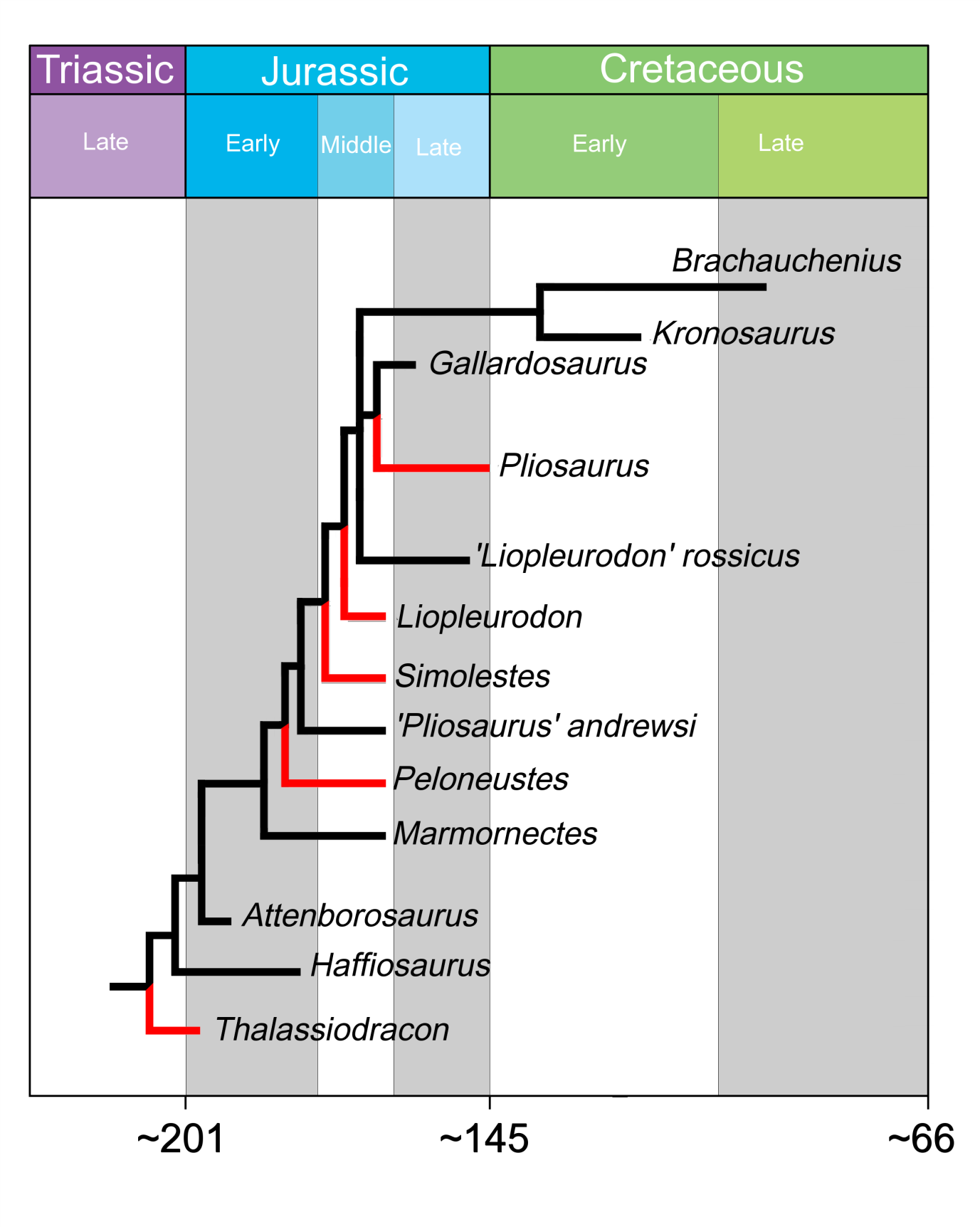


**Figure S1. Simplified phylogenetic tree of Pliosauridae.** Highlighted branches represent taxa used in this study. Phylogenetic relationships follow the results showed in Benson et al. (2013) and Benson & Drukenmiller (2013).
